# SCM, the M Protein of *Streptococcus canis* Binds Immunoglobulin G

**DOI:** 10.3389/fcimb.2017.00080

**Published:** 2017-03-28

**Authors:** Simone Bergmann, Inga Eichhorn, Thomas P. Kohler, Sven Hammerschmidt, Oliver Goldmann, Manfred Rohde, Marcus Fulde

**Affiliations:** ^1^Department of Medical Microbiology, Helmholtz Center for Infection ResearchBraunschweig, Germany; ^2^Institute of Microbiology and Epizootics, Centre for Infection Medicine, Freie Universität BerlinBerlin, Germany; ^3^Department Genetics of Microorganisms, Interfaculty Institute for Genetics and Functional Genomics, Ernst-Moritz-Arndt Universität GreifswaldGreifswald, Germany; ^4^Department of Infection Immunology, Helmholtz Center for Infection ResearchBraunschweig, Germany; ^5^Central Facility for Microscopy, Helmholtz Center for Infection ResearchBraunschweig, Germany

**Keywords:** zoonosis, *Streptococcus canis*, M protein, Immunoglobulin G, anti-phagocytic activity

## Abstract

The M protein of *Streptococcus canis* (SCM) is a virulence factor and serves as a surface-associated receptor with a particular affinity for mini-plasminogen, a cleavage product of the broad-spectrum serine protease plasmin. Here, we report that SCM has an additional high-affinity immunoglobulin G (IgG) binding activity. The ability of a particular *S. canis* isolate to bind to IgG significantly correlates with a *scm*-positive phenotype, suggesting a dominant role of SCM as an IgG receptor. Subsequent heterologous expression of SCM in non-IgG binding *S. gordonii* and Western Blot analysis with purified recombinant SCM proteins confirmed its IgG receptor function. As expected for a zoonotic agent, the SCM-IgG interaction is species-unspecific, with a particular affinity of SCM for IgGs derived from human, cats, dogs, horses, mice, and rabbits, but not from cows and goats. Similar to other streptococcal IgG-binding proteins, the interaction between SCM and IgG occurs via the conserved Fc domain and is, therefore, non-opsonic. Interestingly, the interaction between SCM and IgG-Fc on the bacterial surface specifically prevents opsonization by C1q, which might constitute another anti-phagocytic mechanism of SCM. Extensive binding analyses with a variety of different truncated SCM fragments defined a region of 52 amino acids located in the central part of the mature SCM protein which is important for IgG binding. This binding region is highly conserved among SCM proteins derived from different *S. canis* isolates but differs significantly from IgG-Fc receptors of *S. pyogenes* and *S. dysgalactiae* sub. *equisimilis*, respectively. In summary, we present an additional role of SCM in the pathogen-host interaction of *S. canis*. The detailed analysis of the SCM-IgG interaction should contribute to a better understanding of the complex roles of M proteins in streptococcal pathogenesis.

## Introduction

*Streptococcus* (*S*.) *canis* is an opportunistic pathogen which colonizes the mucosal surfaces and skin of a variety of different animal species. Domestic carnivores, such as dogs and cats, constitute the main hosts, but *S. canis* has also been found in other mammalian species such as rats, minks, mice, rabbits, and foxes (Corning et al., 1991; Iglauer et al., 1991). Predisposing conditions, such as age, underlying diseases, or immunodeficiencies, can lead to infection caused by *S. canis*. Clinical manifestations range from localized infections of skin and mucosa to severe and life-threatening diseases, such as streptococcal toxic shock syndrome, necrotizing fasciitis, septicemia, and meningitis (DeWinter and Prescott, 1999; Kruger et al., 2010; Lamm et al., 2010). Very recently, *S. canis* has also been characterized as a zoonotic pathogen, based on reports of transmission resulting from close contact between humans and dogs and from bite injuries (Bert and Lambert-Zechovsky, 1997; Galperine et al., 2007; Lam et al., 2007; Takamura et al., 2009; Lacave et al., 2016; Tan et al., 2016). However, human infections with *S. canis* do not necessarily require contact with dogs or cats (Amsallem et al., 2014).

The emerging resistances of bacteria against one or multiple antibiotics demands scientific efforts to develop alternative therapeutic approaches against infectious agents. A specific intervention in bacterial-host interactions is a promising approach, but requires profound knowledge of the molecular bases of bacterial virulence traits. However, with only very few exceptions, there is scant knowledge about the virulence mechanisms of *S. canis*. Yang et al. (2010) identified a protein with homologies to the Streptococcal Protective Antigen (SPA) of *S. pyogenes* and *S. equi*, which facilitates protection in a murine infection model after passive immunization (Yang et al., 2010). Hitzmann and co-workers described the Arginine Deiminase System in *S. canis*, which confers resistance against acidic conditions, as found in the host cell phagolysosome (Hitzmann et al., 2013). Finally, we previously identified a surface-associated protein with structural and functional, but not genetic similarities to the M protein family of streptococci, which we therefore designated SCM for *S. canis* M protein (Fulde et al., 2011).

SCM is a fibrillar protein which is found in the majority of *S. canis* isolates (Verkuehlen et al., 2016). The protein exhibits a molecular weight of ~45 kDa and contains heptameric sequence repeats with hydrophobic amino acids at position 1 and 4 leading to an almost alpha helical protein structure with a high tendency for dimerization. Functionally, SCM constitutes a bacterial receptor for host plasminogen (Plg) with a particular affinity for the cleavage product mini-Plg. Plg is a zymogen for the broad-spectrum serine protease plasmin. Although *S. canis* is not able to intrinsically activate Plg, plasmin can be immobilized onto the bacterial surface via SCM, where its enzymatic activity can degrade fibrinogen and aggregate fibrin thrombi (Fulde et al., 2011, 2013a,b). Plasmin mediated tissue degradation is an important virulence trait of disseminative streptococcal infections (Sun et al., 2004).

A major characteristic of streptococcal M proteins is their anti-phagocytic potential. The underlying mechanisms are versatile and include the demasking of bacterial antigens by host-derived extracellular matrix or serum proteins, circumventing the host's complement attack by binding to complement inhibitors or enlarging the bacterial surface by M protein mediated self-aggregation (Whitnack and Beachey, 1982; Horstmann et al., 1988; Kotarsky et al., 1998; Frick et al., 2000; Berggard et al., 2001; Morfeldt et al., 2001; Fulde et al., 2013a). A well-characterized anti-phagocytic immune evasion mechanism of streptococcal M and M-like proteins is their interaction with immunoglobulins. Immunoglobulins (Igs) constitute the main arm of the adaptive humoral immune response. Certain Ig- subtypes, such as immunoglobulin G (IgG), mediate an anti-bacterial immune mechanism referred to as opsono-phagocytosis. Opsono-phagocytosis is a bipartite process, consisting of an antigen-specific interaction between the F(ab) part of IgG and the bacterium, and an interaction between the constant fragment crystallizable (Fc) part of IgG with a specific Fc-receptor, which is usually localized on professional phagocytic immune cells, such as macrophages and granulocytes. IgG-Fc receptors *per se* are versatile and widespread among bacterial species, irrespective of their pathogenicity, metabolism, or their cell wall composition (Forsgren and Sjöquist, 1969; Bjorck and Kronvall, 1984; Zav'yalov et al., 1996; Meehan et al., 2001, 2009; Lewis et al., 2008; Leo and Goldman, 2009). In streptococci, however, non-immune binding to the IgG-Fc region is mediated exclusively by members of the M and M-like protein families, such as protein G and FOG (the fibrinogen binding protein of group G streptococci) of *S. dysgalactiae*sub. *equisimilis* (SDSE), protein H and different M proteins of *S. pyogenes* (Group A streptococci, GAS), SzM of *S. equi* subsp. *zooepidemicus*, and the fibrinogen binding protein (FgBP) of *S. equi* subsp. *equi* (reviewed in Nobbs et al., 2009).

In this study, we describe SCM, the M protein of *S. canis*, as a novel IgG-binding protein, which specifically interacts with the constant Fc part in a non-opsonic manner. This work represents a comprehensive study on molecular and biochemical mechanisms, which characterizes the IgG-SCM interaction in detail and finally helps to expand our knowledge about streptococcal IgG-Fc receptors.

## Methods

### Bacterial strains and growth conditions

*Streptococcus canis* strains used in this study have previously been described (Fulde et al., 2011, 2013a; Verkuehlen et al., 2016). Additional isolates (5539-1-10, 5480-2-10, 5468-1-10, and 5520-2-10) were kindly provided by Dr. Jutta Verspohl (University of Veterinary Medicine Hannover). Where indicated, the *scm*-genotype was evaluated by PCR essentially as described earlier (Verkuehlen et al., 2016). Generation of a *S. gordonii* strain heterologously expressing SCM and its SCM-negative parental strain were described in a prior study (Fulde et al., 2011). SDSE strain G45 and its FOG-negative deletion mutant G89 were described earlier (Nitsche-Schmitz et al., 2007). Bacteria were routinely grown in tryptic soy broth (TSB) at 37°C without shaking. *Escherichia coli* strains were cultivated in Luria-Bertani-Medium. Where indicated, antibiotics were supplemented in the following concentrations. *E. coli*: Ampicillin (100 μg/ml), kanamycin (25 μg/ml); *S. gordonii*: Erythromycin (1 μg/ml).

### Proteins and sera

Human IgG, different subclasses of human IgG, and papain-treated fragments of human IgG as well as human plasminogen were purchased from Sigma. C1q was purchased from Calbiochem. Polyclonal antibodies against human plasminogen and secondary, HRP-conjugated anti-rabbit, anti-mouse, and anti-human antibodies were purchased from Dako.

### Radioactive labeling, binding, and inhibition studies

Iodination of IgG, Plg, and SCM was performed according to the chloramine T method essentially as described earlier (Chhatwal et al., 1987). Binding and competition studies were carried out as described in Fulde et al. (2011, 2013a).

### Flow cytometry analyses

Bacteria were grown to mid-exponential phase in TSB medium, sedimented by centrifugation at 1,400 × g for 10 min and washed once with 10 ml phosphate buffered saline (PBS). A total of 5 × 10^7^ bacteria in 100 μl of PBS containing 0.5% fetal calf serum (FCS) and incubated with 50 μg/ml polyclonal rabbit IgG for 30 min at 37°C. After washing the bacteria two times with PBS, the bacterial pellet was suspended in 100 μl of PBS containing a 1:250 dilution of an anti-rabbit ALEXA® Fluor 488 antibody or an anti-rabbit ALEXA® Fluor 568 respectively and incubated for 45 min at 37°C. Bacteria were again washed two times with 1 ml PBS, fixed by adding 3% paraformaldehyde in PBS followed by flow cytometry analysis using a FACSCalibur (Becton Dickinson). Streptococci were detected using log-forward and log-side scatter dot plots, and a gating region was set to exclude larger aggregates of bacteria. A total of 10^4^ bacteria were analyzed for fluorescence using log-scale amplification. Histograms of representative results are shown.

### Cloning and expression of truncated SCM fragments

Using plasmid *p*QE30-SCM (Fulde et al., 2011) as the parental construct, a technique for generation of truncated fragments was developed using inverse PCR-amplification. Depending on the region of interest (C-terminal or N-terminal) oligonucleotides were designed annealing either in the His-tag region or downstream of the stop codon. An appropriate internal oligonucleotide was designed for specific N-terminal or C-terminal truncations, respectively. SCM KO173225, a fragment with an internal deletion was generated by a combination of internal oligonucleotides. A diagram illustrating the cloning strategy is depicted in Supplementary Figure 1. Oligonucleotides used in this study harbor a terminal *Eco*RV restriction site and are listed in Table 1. The N-terminal 37 amino acids were removed from recombinant SCM fragments, in order to enable purification of the proteins out of the *E. coli* cytoplasm as described below.

**Table 1:** **Oligonucleotides used in this study**.

**Fragment**	Oligonucleotide: 5′ → 3′^*^
N-173	gggqatatc TCTTGGCT AGCAGATAGTTGTGC
	cccqatatcTGAGTCGACCTGCAGCCAAGC
N-207	gggqatatc GCAGAAATTGAGCGCTTGACA
	cccqatatc TGAGTCGACCTGCAGCCAAG
N-225	qggqatatc CACTTCTGCTTGAAGTTTCTCG
	cccqatatcTGAGTCGACCTGCAGCCAAG
C-67	cccqatatc TCGAAAAGAAGTGAAA T A TTT AG
	gggqatatc TCCGTGATGGTGATGGTGATGCGATCC
C-90	cccqatatcTCATACTGATATGATTGAAAAAGAG
	qggqatatcTCCGTGATGGTGATGGTGATGCGATCC
N-275	qggqatatcAGCTACTTT AGCTTGCTCTGCGGC
	cccgatatcTGAGTCGACCTGCAGCCAAG
C-112	cccqatatc T AA T AAAGGTCTT ACT AAGG
	gggqatatc TCCGTGATGGTGATGGTGATGCGATCC
C-142	cccqatatcAGCT AACCTTGACGCTTTGAACC
	qggqatatcTCCGTGATGGTGATGGTGATGCGATCC
C-173	cccqatatc GAGCGT AACGCTGAGTT AGAGCG
	qgggatatcTCCGTGATGGTGATGGTGATGCGATCC
C-202	cccqatatcAGAAATTGAGCGCTTGACAGCTG
	qggqatatcTCCGTGATGGTGATGGTGATGCGATCC
C-226	cccgatatcTCAACACTTCGTGATCAAGTAGCAAGCC
	gggqatatcTCCGTGATGGTGATGGTGATGCGATCC
K0173225	gggqatatc TCTTGGCT AGCAGATAGTTGTG
	cccqatatcTCAACACTTCGTGATCAAGTAGCAAGCC
*p*QE30-Seq 5′	CCCGAAAAGTGCCACCTG
*p*QE30-Seq 3′	GTTCTGAGGTCATTACTGG

* *EcoRV restriction site is underlined*.

The inverse PCR was performed using the Phusion® High-Fidelity DNA Polymerase (Finnzymes) following the manufacturer's recommendations with the following parameters: Initial denaturation: 98°C, 30 s, denaturation: 98°C, 10 s, annealing: 60°C, 30 s, elongation: 72°C, 180 s, final elongation: 72°C, 600 s. PCR amplificates were digested with *Eco*RV and re-ligated using T4 DNA-Ligase (Promega) and subsequently transformed into electrocompetent *E. coli* M15[*p*REP4]. Protein expression was performed using the lactose-inducible expression system of the vector *p*QE30 in the *E. coli* host strain M15[*p*REP4] as described by the manufacturer (Qiagen Expressionist™ System). In brief, 400 ml of a bacterial culture was inoculated with the recombinant *E. coli* clone and cultured at 37°C with aeration (shaking flasks at 280 rpm) until an optical density at 600 nm of 0.6 (OD600). Protein expression was induced by incubation with 1 mM of the lactose analogon isopropyl-ß-D-thiogalactoside (IPTG) for 4 h at 28°C and 140 rpm shaking. After sedimentation of the bacteria at 3,000 × g at 4°C, the sediment was resuspended in 10 ml PBS and mechanically lysed using a French Press (SLM Instruments). After additional centrifugation at 10,000 × g for 30 min at 4°C, the supernatant was applied onto a prepacked and equilibrated Protino NED 2000 Ni-NTA column (Macherey-Nagel) for purification of His-tagged proteins by affinity chromatography. The proteins were eluted from the Ni-NTA-columns by addition of 250 mM imidazole according to the recommendations of the manufacturer. After dialysis of the eluted protein against PBS, the protein concentrations were determined using Bradford assays (Serva). Prior to binding studies, the quality of the truncated proteins (presence of a single protein band, no degradation products) was verified by SDS-PAGE.

### Radioactive and non-radioactive dot blot analysis

Radioactive Dot Blot analysis was carried out essentially as described in Fulde et al. (2011). Briefly, IgGs derived from different host species as well as different subclasses of human IgG were spotted onto a nitrocellulose membrane in a concentration series. The membrane was blocked with 5% skimmed milk at 4°C overnight. The following day, the membrane was washed five times by moderate shaking for 10 min at RT with 10 ml PBS supplemented with 0.05% Tween-20 (PBST). Hybridization with ^125^I-SCM was performed for 4 h at room temperature (RT). Hybridization (binding) was detected after exposure of X-ray films (Kodak).

Non-radioactive Dot Blot analyses were performed for determination of the minimal IgG interaction sites of the SCM protein. Truncated fragments of SCM were immobilized by spotting onto nitrocellulose membranes. Blocking and washing procedures were performed as described previously (Fulde et al., 2011). HRP-conjugated rabbit anti-goat antiserum was applied as a ligand for interaction studies. Binding was detected by chemiluminescence determination after incubation of the blots with Pierce® ECL Western Blotting substrate according to the recommendations of the manufacturer. Signal development was performed by exposure of Amersham ECL-Hyperfilm (GE Healthcare) followed by an automated development with a Typon C2 developer (NDTMED-Röntgentechnik).

### Elisa

In addition to Dot Blot analyses, characterization of the IgG-binding site of SCM was performed with ELISA. Different concentrations of full-length or truncated SCM fragments (100, 50, 25, 12.5 μg/ml) were immobilized in a 96-well plate with 0.1 M NaHCO_3_ at pH 9.6. After blocking for 2 h at RT with 1% BSA in PBST and three wash steps with PBST using a volume of 100 μl, a 1:5000 dilution of polyclonal HRP-conjugated rabbit anti-goat antiserum was added for 2 h. IgG- incubation without proteins was used as negative control. Signal determination was performed with 50 μl of tetramethyl bencidine substrate solution (TMB, Thermo Fisher Scientific) as recommended by the manufacturer. The enzyme reaction was stopped after 15 min at RT by adding 50 μl of 2 N H_2_SO_4_ and the color reaction was measured using an ELISA reader (Tecan Sunrise) at 450 nm (reference wave length 570 nm). Experiments were repeated three times using triplicate samples.

Competitive IgG binding studies have been carried out with SCM and FOG, the M protein of *S. dysgalactiae* sub. *equisimilis*, which harbors fibrinogen and IgG binding capability (Johansson et al., 2004; Nitsche-Schmitz et al., 2007). In these assays, 10 μg of SCM, FOG, and the truncated SCM-fragment KO173225, which lacks the internal IgG-binding region, were pre-incubated with human HRP-conjugated IgG (1:200 dilution) for 15 min. Wells of a 96**-**well plate were coated with 5 μg of full lengths SCM protein in 0.1 M NaHCO_3_, pH 9.6. After blocking for 2 h at RT with 1% BSA in PBST followed by three wash steps with PBST, pre-incubated mixtures of bacterial proteins with IgG were added to the SCM-coated wells and incubated for 2 h. IgG without proteins was used as negative control in various concentration. Signal determination was performed after incubation for 30 min with 50 μl of activated 2,2′-Azino-di-(3-ethylbenzthiazolin)-6-sulphonic acid (ABTS). The ABTS solution was prepared by adding 100 μl of 3% H_2_O_2_ to 11 ml of substrate solution directly before development reaction. The enzyme reaction was stopped by adding 50 μl of 2N H_2_SO_4_. Signals were determined with an ELISA reader (Tecan Sunrise) at 405 and 655 nm as reference. Experiments were repeated three times using triplicate samples.

### Detection of C1q immobilization on the streptococcal surface

Detection of C1q binding was performed according to the protocol of Nitsche-Schmitz and colleagues with slight modifications (Nitsche-Schmitz et al., 2007). Briefly, overnight cultures of *S. canis* strain G361 (SCM^+^) and G2 (SCM^−^) as well as SDSE strain G45 (FOG^+^) and G89 (FOG^−^) grown in Todd Hewitt liquid medium (Difco) at 37°C and 5% CO_2_ were sedimented by centrifugation at 1,400 × g for 10 min and washed with PBS supplemented with 0.05% Tween-20 (PBST) and resuspended in 3 ml of PBS in a concentration of 0.25 g of bacteria/ml (wet weight/volume). Hundred microliters of this suspension was incubated with 10 μg of human IgG (Sigma) for 30 min at RT. Another 100 μl of the bacterial suspension was incubated with 2 μg purified C1q (Calbiochem) for 1 h at 37°C, and a third fraction was pre-incubated for 30 min at RT with 10 μg IgG. After two wash steps with 1 ml PBS, 2 μg of C1q was added to the pre-incubated suspension and incubated at 37°C for 1 h. Then, bacteria were washed three times with PBS, and surface-bound proteins were eluted by incubation with 30 μl of 100 mM glycine, pH 2.0 for 15 min at RT followed by centrifugation for 10 min at 1,400 × g. Samples were neutralized by addition of 1.5 M Tris-HCl, pH 8.8. Proteins were separated by SDS-PAGE and blotted onto a nitrocellulose membrane. Subsequent Western Blot analysis was performed essentially as described (Nitsche-Schmitz et al., 2007).

### Field emission scanning electron microscopy (FESEM) for visualization of bound IgG

*S. canis* strain 361, G15, G17 and strains G2 and 2424/96 were incubated with a 1:50 dilution in PBS of protein A-purified IgG protein (stock solution 1,7 mg/ml) of pre-immune serum of rabbit for 1 h at 37°C. Bound IgG was visualized by incubation with protein A-gold/protein G-gold nanoparticles (ratio 1:1, 15 nm in size) for 30 min at 37°C. After washing with PBS and TE buffer (10 mM TRIS, 2 mM EDTA, pH 6,9), samples were placed onto butvar coated copper grids and air dried. Grids were attached to adhesive carbon tape on aluminum stubs and samples were examined in a Zeiss Merlin (Oberkochen, Germany) applying the Everhart-Thornley HESE2-secondar electron detector at an acceleration voltage of 10 kV. Images were taken with the Zeiss SmartSEM software version 5.05. Contrast and brightness were adjusted with Adobe Photoshop CS5.

### SCM phylogeny

Genomic DNA of 46 *S. canis* strains (Verkuehlen et al., 2016) was used for PCR amplification of the IgG-binding region of SCM using DreamTaq™ DNA Polymerase (ThermoScientific) with DreamTaq green PCR Master Mix (ThermoScientific) according the manufacturers protocols with the following oligo-nucleotides: SCM_bind_FP (5′–3′ ATC TGC TAG CCA AGA GCG TA) and SCM_bind_RP (5′–3′ CAC TTC TGC TTG AAG TTT CT) and the following temperature parameters: Initial denaturation: 94°C, 180 s, denaturation: 94°C, 180 s, 30 cycles of annealing: 58°C, 30 s, elongation: 72°C, 30 s, final elongation: 72°C, 600 s. The obtained PCR-products were Sanger-sequenced by LGC Genomics Company, Germany, which generated high-quality sequences of approximately 169 bp. The sequences of the IgG-binding region of SCM were aligned using the Geneious Alignment with default parameters provided by the Geneious 7.0.2 software. The identified sequence variants were used as template for a megablast search against the NCBI nucleotide database in order to identify published reference sequences for each sequence variant. Examples of each variant of the IgG-binding region of SCM of the 46 *S. canis* strains, their identified reference sequences as well as the IgG-binding region of SCM of G1, G8, G13, G15, and G361 were aligned with the Geneious Alignment and were used to generate a UPGMA tree using Geneious Tree Builder with a Jukes-Cantor distance model.

M-like proteins of other streptococcal species were identified using blastp with the protein sequence of the IgG-binding region of SCM as query target against the NCBI protein database. The identified protein sequences were aligned with Geneious Alignment with a Blosum62 cost matrix and the resulting alignment further used to generate a UPGMA tree using Geneious Tree Builder with a Jukes-Cantor distance model.

### Surface plasmon resonance (SPR)

Protein-protein interactions between SCM-WT or KO173225 and human IgG-Fc were analyzed by SPR using a BIAcoreT100 optical biosensor (GE Healthcare). Recombinant SCM and KO173225 were immobilized as ligands on a carboxymethyl dextran (CM5) sensor chip using standard amine-coupling procedures as described earlier (Kohler et al., 2015). Briefly, SCM and KO173225 proteins were adjusted to 10 μg/ml in 10 mM acetate buffer (pH 4.0) and injected for surface immobilization at a flow rate of 10 μl/min, followed by deactivation of residual activated groups with 1 M ethanolamine. The resonance values of the bound proteins (RU) reached ~2,200 RU. The control flow cell was prepared identically but without protein injection. Binding analysis was performed with PBS containing 0.05% Tween®-20 (PBST) at 25°C using a flow rate of 10 μl/min. Regeneration of the affinity surface was carried out with 10 mM glycin pH 3.0. The given resonance units (RU) in the sensograms represent the RU values after subtraction of the values measured in the blank chamber. Each interaction was measured at least three times. Data were analyzed using BIAcoreT100 Evaluation Software (version 2.0.1.1). Experimental data were fitted globally using the simple one-step bimolecular association reaction (1:1 Langmuir kinetic: A+B↔AB).

### Statistical analyses

Unless stated otherwise, all experiments were repeated three times using triplicate samples and results are presented as mean values including standard deviations. Significance was calculated using an one-way ANOVA followed by Dunnett's post**-**test. *P*-values < 0.05 are considered as significant. Significant differences in data sets are indicated with an asterisk.

## Results

### The *streptococcus canis* M protein SCM is a bacterial receptor for immunoglobulin G

**Figure 1:**
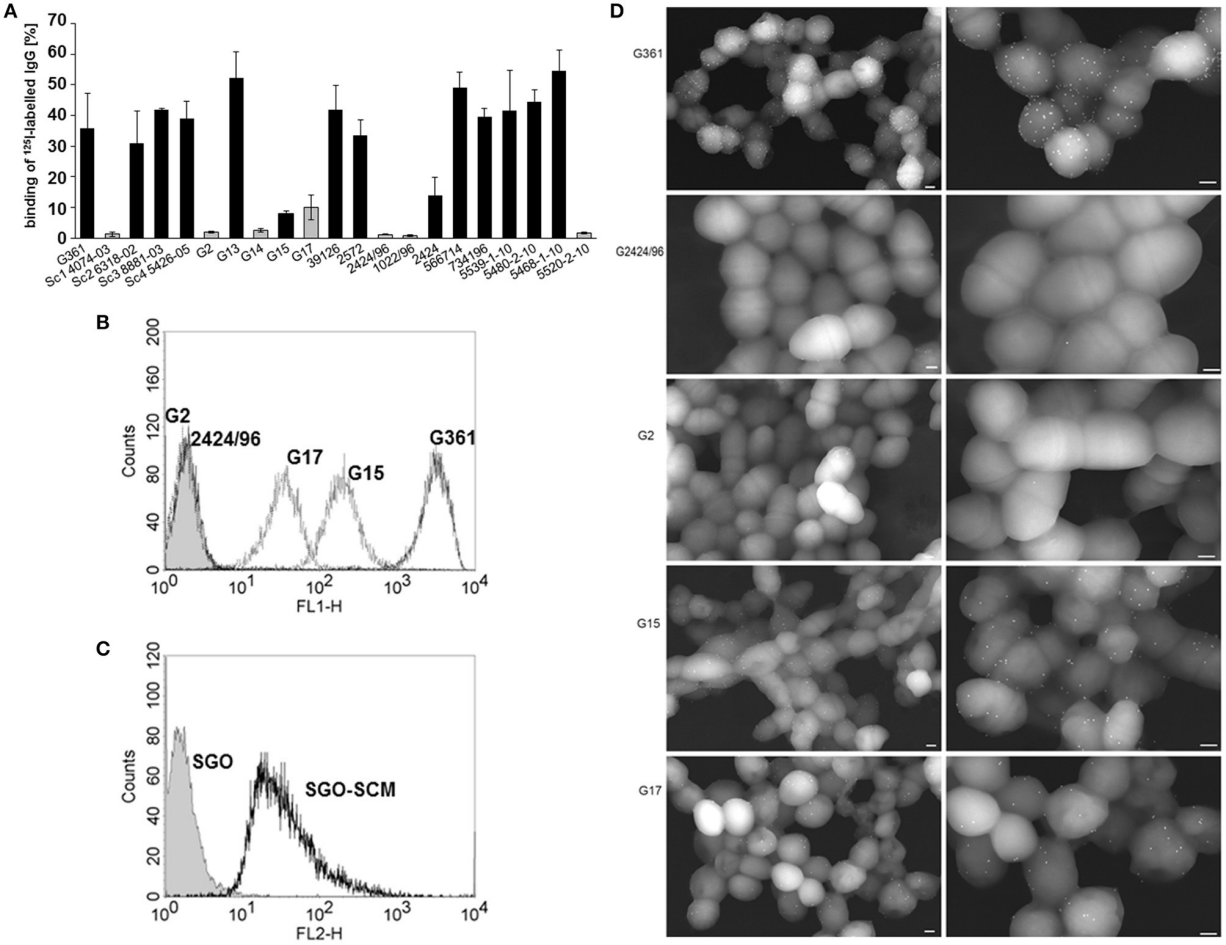
**IgG binding to *S. canis*. (A)** Binding of iodinated IgG to 21 different *scm*-positive (black bars) and *scm*-negative (gray bars) *S. canis* isolates was analyzed by gamma-counting. Data represent triplicates of three independent experiments and are presented as mean values ± standard deviation. IgG-binding to *scm*-negative *S. canis* G2 and G2424/96, and G17, and to SCM-expressing *S. canis* G361 and G15 **(B)** as well as to *S. gordonii* bacteria (SGO) and heterologously SCM-expressing *S. gordonii* bacteria **(C)** was analyzed by flow cytometry using polyclonal IgG raised in rabbit and fluorescence—conjugated secondary antibody. IgG binding was detected for SCM-expressing *S. canis* strains and also for SCM-expressing *S. gordonii*. Histograms of representative results are shown. **(D)** Field Transmission Electron microscopic visualization of the bacterial strains G361, G2424/96, G2, G15, and G17. Bound IgG was detected with protein A-gold nanoparticles. FESEM analysis of *S. canis* G2 and G2424/96 revealed only marginal background binding, whereas strong IgG binding is shown for G361 and moderate binding was visualized on the surface of G15 and G17 (white spheres). Bars represent 200 nm.

We recently identified SCM as an M protein of *S. canis*, characterized by its affinity for the serum protein plasminogen and its anti-phagocytic properties (Fulde et al., 2011, 2013a,b). In the present study, we were interested in determining whether the SCM protein might possess additional binding activities to other serum proteins with immune-modulatory functions, in particular immunoglobulin G. We, therefore, incubated different SCM-positive and-negative *S. canis* strains with iodinated rabbit IgG. IgG binding was detected for the majority of isolates with percentages ranging from 13.93 ± 5.89% (strain 2,424) to 54.64 ± 6.76% (strain 5468-1-10; Figure 1A). Strains Sc1 4074-03, G2, G14, G15, G17, 2424/96, 1022/96, and 5520-2-10 showed binding efficiencies lower than 10% and were classified as non-IgG binders. Interestingly, with the exception of strain G15 (8.09 ± 0.82% IgG binding), all non-IgG binding isolates harbor an *scm*-negative genotype in the recently described all-*scm* PCR (Fulde et al., 2011; Verkuehlen et al., 2016). These data strongly suggested a role for SCM as an IgG receptor in *S. canis*. To verify this hypothesis, the SCM-positive *S. canis* strains G15 and G361 and the SCM-negative strains G2, G17, and 2424/96 were incubated with Alexa 488-conjugated rabbit-anti goat antibody (diluted 1:500 in PBS) and subjected to FACS analysis. As expected, fluorescence intensity of strain G361 was substantially increased in contrast to the SCM-negative strains G2 and 2424/96, respectively (filled curves, Figure 1B). Interestingly, an intermediate phenotype was detected for the SCM-positive strain G15 and the SCM-negative strain G17. The role of SCM as an IgG receptor was further confirmed by flow cytometric analysis of *S. gordonii* bacteria (SGO-SCM) heterologously expressing SCM on their surface. As shown in Figure 1C, a substantial shift in fluorescence intensity was observed for strain SGO-SCM but not for its SCM-negative parental strain SGO-WT. These results clearly demonstrated a role for *S. canis* SCM in IgG binding. To further confirm the results from the binding studies with iodinated IgG and FACS analysis, we visualized IgG binding by field scanning electron microscopy. As depicted in Figure 1D, a high amount of 15 nm dots (resembling gold-labeled IgGs) was observed on the surface of SCM-positive strain G361, whereas the SCM-negative strains G2 and 2424/96 were free of any electron-dense particles (Figure 1D). Again, strains G15 and G17 exhibit an intermediate phenotype with a reduced amount of surface-bound IgG molecules.

### Phenotypic characterization of the SCM—IgG interaction

Since *S. canis* has been characterized as a zoonotic agent able to cause infections in a variety of different hosts, we were interested in the interaction of SCM with IgG derived from different species. Using Dot Blot analysis with iodinated SCM as the ligand, a specific signal was detected for IgGs derived from cats, dogs, horses, human, and rabbits (Table 2). Surprisingly, although *S. canis* is frequently isolated from mastitis in ruminants (Chaffer et al., 2005; Hassan et al., 2005), neither an interaction with bovine nor with goat IgG was observed. In order to determine (putative) bacterial species-specific differences, we then incubated SCM with the different subtypes (IgG1, IgG2, IgG3, and IgG4) of human IgG. However, as already shown for the M protein FOG of SDSE (Nitsche-Schmitz et al., 2007), a direct binding of SCM to IgG1, IgG2, and IgG4 but not to IgG3 was observed (Table 2). Finally, this interaction is non-opsonic, since Dot Blot analysis with iodinated SCM revealed a specific signal for the conserved IgG-Fc part, but not for IgG-Fab (Table 2). In summary, our data demonstrate a species-specific, non-opsonic and potentially immune-modulatory mode of SCM-IgG interaction.

**Table 2:** **Specificity of IgG-SCM interaction**.

**Species**	**Binding**
Dog	+
Cat	+
Cattle	–
Horse	+
Human	+
Mouse	+
Goat	–
Rabbit	+
**hlgG-subclasses**	**Binding**
lgG1	+
lgG2	+
lgG3	–
lgG4	+
**hlgG-fragments**	**Binding**
F_c_	+
Fab	–

Iodinated SCM was applied as ligand in Dot Blot overlay analyses with immobilized IgG derived from different species, with IgG subclasses 1–4 and IgG Fab- and Fc- fragments. A positive signal was indicated with “+,” no binding was indicated with “–.”

### Concurrent and non-inhibitory binding of IgG and plasminogen to SCM

**Figure 2:**
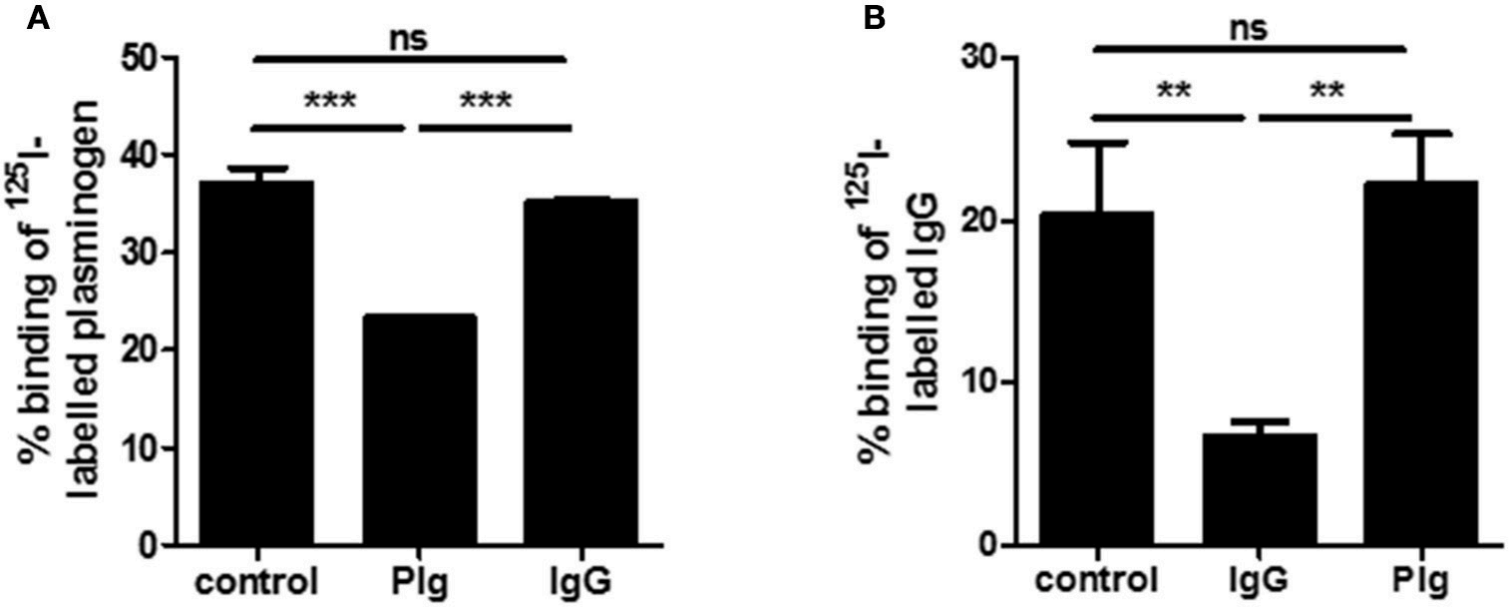
**Plasminogen and IgG binding of SCM**. In radioactive inhibition studies binding of iodinated plasminogen **(A)** or iodinated IgG **(B)** to *S. canis* G361 was determined after incubation with non-labeled IgG (1.0 μM) or plasminogen (1.0 μM). Data represent triplicates of three independent experiments and are presented as mean values ± standard deviation. Significance was calculated using an one-way ANOVA followed by Dunnett's post**-**test. ^**^P-values < 0.01, ^***^P-values < 0.001.

In prior studies, we had identified plasminogen (PLG) as a ligand for SCM (Fulde et al., 2011, 2013a,b). The detection of IgG as an additional binding partner for SCM inevitably raised the question as to whether PLG and IgG compete for the same binding site on SCM. To answer this question, we incubated the SCM-positive *S. canis* strain G361 with iodinated IgG. As already shown in Figure 1A, a binding capacity of ~20.4 ± 4.5% was detected (Figure 2A). Excess, non-iodinated IgG (1 μM) showed a significant inhibition of IgG-binding to ~6.7 ± 0.9%. In contrast, addition of 1 μM of plasminogen did not alter IgG-binding capacity of strain G361 (22.3 ± 3.1%, Figure 2B). Similarly, incubation in the presence of excessive non-radiolabeled plasminogen to *S. canis* strain G361 PLG decreased iodinated PLG-binding capacity from 37.1 ± 1.5% to ~23.3 ± 0.2. As expected, supplementation of non-labeled IgG did not alter the PLG-binding capacity of G361 (Figure 2A). These results clearly demonstrated a concurrent binding of IgG and PLG to SCM, which occurs in a non-competitive manner.

### The IgG-binding site of SCM is located in the central part of the mature protein

**Figure 3:**
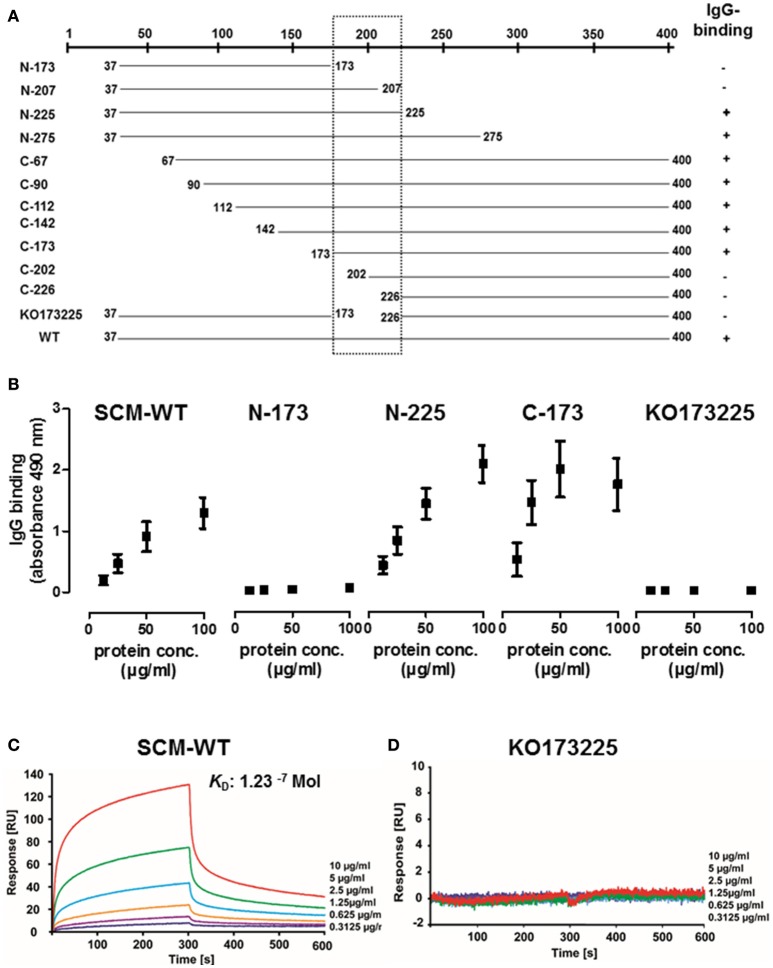
**Identification of the specific IgG-binding site in SCM of *S. canis* G361. (A)** Summary of ELISA and quantitative Dot blot assays to study IgG-binding activity by various C-terminal, N-terminal, and truncated SCM fragments. Positions of first and last amino acid of the fragments are indicated. IgG binding activity is indicated with “+.” **(B)** The truncated N-terminal SCM fragments N-173 and N225, the C-terminal fragment C-173, and a fragment lacking the putative IgG-binding region ranging from amino acid 173 to 225 were tested for IgG-binding activity by ELISA. **(C,D)** Interactions of soluble human IgG-Fc with immobilized SCM-WT or KO173225 were analyzed by surface plasmon resonance spectroscopy. The representative sensograms show a dose-dependent binding of IgG-Fc to SCM-WT whereas no binding was detectable for KO173225. The association and dissociation was observed, each of 300 s. Values of the control flow cells were subtracted from each sensogram. The K_*D*_ value is indicated.

In order to identify binding site(s) for IgG, numerous C-terminal, N-terminal, and truncated deletion fragments of SCM were generated and tested for their IgG-binding activity using quantitative Dot Blot assays and ELISA. The N-terminal SCM fragments N-225 and N-275 revealed a strong IgG-binding signal (Figure 3B, Supplementary Figures 2, 3). Reducing the length of the truncated fragment to 136 amino acids (N-173), completely abolished the IgG-binding. Generation of C-terminal fragments by deletion of N-terminal amino acids enabled the detection of positive binding for SCM fragments C-67, C-90, C-112, C-142, and C-173, whereas, in accordance to the N-terminal fragments, C-226 was negative for IgG binding (Figure 3B, Supplementary Figures 2, 3). A deletion construct of mature SCM which lacks a stretch of amino acids 173–225 (KO173225) was also negative for IgG binding, defining this stretch of 52 amino acids located in the central part of the mature SCM as important for IgG binding (Figure 3A).

The surface plasmon resonance (SPR) technique is a robust and valid method to determine quality of protein-protein interactions. To further define the interactions between SCM and IgG, we immobilized SCM-WT on a CM5 sensor chip as a ligand and applied human IgG-Fc in a series of concentrations (0.3–10 μg/ml) as the analyte. The sensogram depicted in Figure 3C shows a specific and dose-dependent binding of IgG-Fc to SCM. A global fitting of response data according to the 1:1 Langmuir model revealed an accurate data fit with low Chi^2^ -values and indicated a simple 1:1 binding site interaction between IgG Fc and SCM. The calculated dissociation constant for the binding of IgG-Fc to SCM was 1.23^−^^7^ Mol, which indicates a strong and physiologically relevant interaction (Supplementary Figure 5). As a control, we included the deletion fragment KO173225, which was negative for IgG binding in Dot Blot analysis, ELISA, and SPR (Figures 3B,D, Supplementary Figures 3, 5).

### The IgG-binding site of SCM is highly conserved among different *S. canis* isolates and other animal-specific and zoonotic streptococci

**Figure 4:**
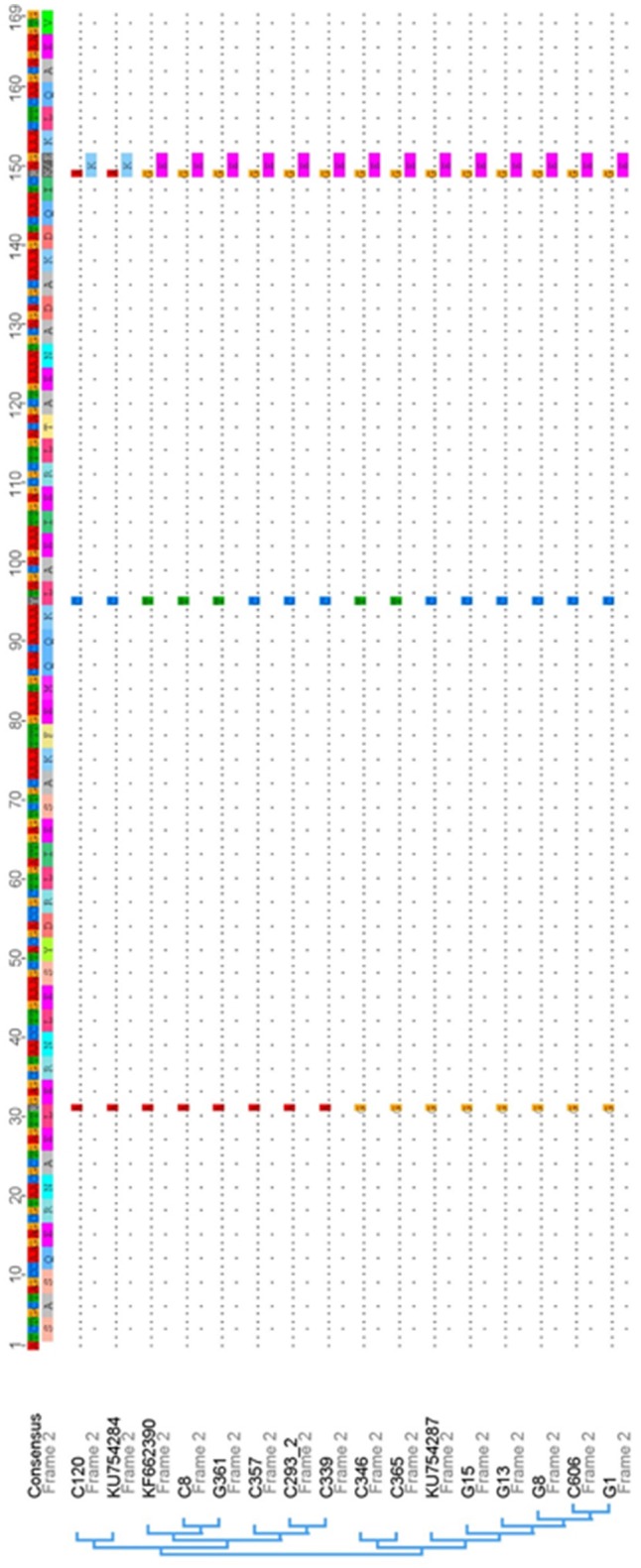
**Nucleotide sequence alignment and phylogenetic analysis of IgG-binding site sequences derived from SCM and other M proteins of *S. canis* strains and their respective references obtained from the nucleotide database of NCBI**. The consensus nucleotide sequence is displayed on top of the alignment followed by the respective amino acid translation. Within the alignment sequence, similarities are displayed with dots and single nucleotide polymorphisms (SNPs) are shown with the regarding nucleotide and amino acid in one letter code, if the SNP is a non-synonymous one. Overall within the displayed IgG-binding region of SCM only three SNPs were detected, with one non-synonymous SNP that is represented by only a single strain and the associated reference sequence.

The close association of the *S. canis* genotype (*scm*-positive) with the ability of the respective isolate to interact with IgG (Figure 1A), strongly suggested that the specific interaction site defined above is highly conserved. To confirm this hypothesis, we generated oligonucleotides which specifically amplified the respective IgG-binding site and tested *scm* positive isolates of a recently published *S. canis* strain collection derived from different hosts (Verkuehlen et al., 2016). In addition, we included the control strains G1, G8, G13, G15, and G361, which were characterized as *scm*-positive, and, with exception of strain G15, exhibited strong IgG-binding activity (Figure 1A). As expected, the homology of the different IgG-binding regions comprising aa 173–225 of the mature protein is high (98.2 to 99.0% identity) with only three SNP sites detected at positions 31 (A or G), 95 (C or T), and 149 (A or G) of the 169 bp nucleotide sequence (Figure 4). The SNP in position 149, represented with nucleotide A, was only detected in one *S. canis* strain (C120) and the reference strain KU754284. This is the only non-synonymous SNP among the detected SNPs, resulting in a lysine (K) instead of glutamic acid (E) as represented by all other sequences. Finally, a mammalian host-species dependent correlation between the nucleotide sequences of bacterial SCM proteins was not observed.

### Analogous, but not homologous IgG-binding of SCM and FOG has different impact on the opsonisation by C1q

**Figure 5:**
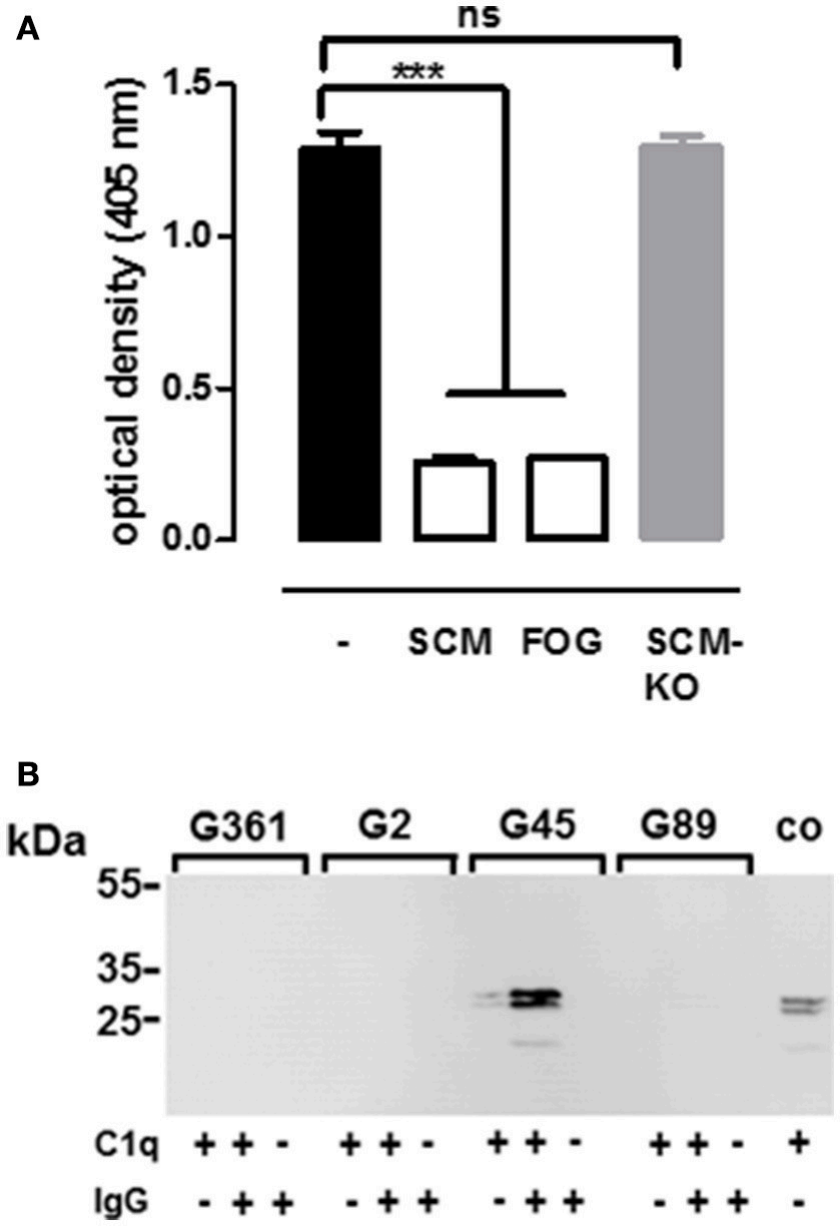
**Analogous rather than homologous interaction of SCM and FOG with IgG-Fc and C1q. (A)** Inhibition of IgG binding to SCM was determined by in ELISA after incubation of 10 μg of M protein FOG, SCM, and the truncated SCM protein fragment KO173225 (SCM-KO). Results represent triplicates of three independent experiments and are presented as mean values ± standard deviation. Significance was calculated using one-way ANOVA followed by a Dunnett's post**-**test. ^***^P-values < 0.001, ns, not significant. **(B)** Different streptococcal strains harboring either a SCM- (G361) or FOG- (G45) positive or a SCM- (G2) and FOG- (G89) negative genotype, respectively, were co-incubated with IgG, C1q, or IgG and C1q in combination. After eluting host proteins form the bacterial surface, immobilization of C1q was determined in Western blot analysis using antibodies directed against C1q. Recombinant C1q served as a positive control (co).

Although the respective IgG-binding regions of SCM and FOG differ significantly (Supplementary Figures 4, 6), both proteins show high similarities in their binding behavior to IgG. For example, both proteins mediate interactions with IgG via a binding site which is located in the central part of the mature M protein, both proteins lack a specific affinity for human IgG3, and both proteins interact with IgG in a non-opsonic manner. This observation prompted us to analyse whether SCM and FOG share a common interaction site on the IgG molecule. We therefore established a competitive ELISA in which recombinant SCM was immobilized in the wells of a 96-well plate. Subsequently, HRP-conjugated human IgG was pre-incubated with 10 μg of recombinant SCM, FOG, or truncated SCM fragment KO173225, respectively, before application to the immobilized SCM protein. As shown in Figure 5A, pre-incubation of IgG with SCM significantly decreased IgG-binding to immobilized SCM to ~20%. Similar results were observed when IgG was pre-incubated with 10 μg FOG. However, as expected, SCM-mediated IgG-binding was not affected when pre-incubated with the truncated SCM fragment KO173225, which lacks the specific IgG-binding site determined in this study.

Nitsche-Schmitz and colleagues had previously demonstrated that, in contrast to protein G, the interaction of the M protein FOG with the Fc region of IgG allows an additional opsonisation with the complement component C1q (Nitsche-Schmitz et al., 2007). Taking the similarities of SCM and FOG in terms of IgG-Fc- binding into account, we hypothesized that C1q is also able to bind to the IgG-SCM complex. For validation, we incubated the SCM-positive *S. canis* isolate G361, the SCM-negative counterpart G2, as well as the FOG-positive SDSE strain G45 and its FOG-negative deletion mutant G89 either with IgG alone, with C1q alone, or with a combination of both IgG and C1q. Bound proteins were eluted from the bacterial surface and analyzed by Western Blot with antibodies directed against C1q. As shown in Figure 5B, neither SDSE nor *S. canis* were opsonized by C1q alone. However, as expected, pre-incubation with IgG led to a FOG-dependent recruitment of C1q to the surface of SDSE. In contrast, C1q was not detected on the bacterial surface of *S. canis*, neither in the SCM-negative nor in the SCM-positive strain. These results indicate that, in addition to the strong phenotypic similarities in IgG-binding by SCM and FOG, the impact on subsequent immune functions, such as the recognition by the complement system, can be highly variable.

## Discussion

Bacteria have evolved numerous strategies to circumvent antibody-mediated killing. An ancestral, but widely distributed mechanism among different bacterial species is the non-immune, anti-opsonic binding of IgGs via their constant fragment crystallizable (Fc) region (Forsgren and Sjöquist, 1969; Bjorck and Kronvall, 1984; Zav'yalov et al., 1996; Lewis et al., 2008; Leo and Goldman, 2009; Meehan et al., 2009). This interaction consists of a simple competition inhibition between the bacterial- and the IgG-Fc receptor expressed on the surface of professional immune cells. In streptococci, the large family of M- and M-like proteins constitutes bacterial IgG-Fc receptors. These proteins share common structural and functional characteristics. For example, M-and M-like proteins are surface-attached fibrillar proteins and usually harbor a modular structure with a homologous C-terminal and a variable N-terminal part. In addition to IgG, M and M-like proteins constitute the receptor for a variety of different host proteins from the serum or the extracellular matrix, such as fibrinogen, plasminogen, complement factors, and IgA (reviewed in Fischetti, 2016). However, their most important feature is a specific anti-phagocytic activity, which has been shown for M proteins, but only suggested for M-like proteins (Metzgar and Zampolli, 2011; Fulde et al., 2013a).

Streptococci expressing M proteins are subtyped by sequence variations in the hypervariable, N-terminal region of the corresponding *emm* genes, resulting in different M protein serotypes (Metzgar and Zampolli, 2011). Although *S. canis* is characterized as *emm*-negative, our group was able to identify SCM, a protein with structural and functional characteristics of M proteins. The anti-phagocytic activity of SCM is based on its particular affinity for plasminogen, which confers a significant protection against phagocytic attacks of polymorphnuclear granulocytes (PMNs) (Fulde et al., 2013a). However, the present study suggests additional roles for SCM with regard to anti-phagocytic properties other than those mediated by plasminogen-binding. As depicted in Figure 1, we clearly demonstrated a strong and reliable correlation between the *scm*-genotype and the IgG-binding phenotype of *S. canis*, with only two exceptions. Isolate G15 is characterized as *scm*-positive using the gene-specific PCR established in our lab (Fulde et al., 2011), but failed to show a substantial IgG-binding capability in radioactive binding assays and an intermediate phenotype in FACS analysis. We consider two explanations for this observation: (i) the particular isolate does not express SCM at all, or (ii) the IgG-specific binding site of the SCM protein is modified. This inconsistency was answered by generating a variety of different truncated SCM fragments to narrow down the IgG-binding site. Finally, we identified a protein stretch of 52 amino acids located in the central part of the mature protein which harbors IgG-binding activity (Figure 3A). Subsequently, nucleotide sequence analyses and sequence comparisons of *scm* alleles derived from a variety of *S. canis* strains revealed a high homology with only three SNPs occurring in the entire IgG-binding region. Using strain G361 as the reference strain, two of those SNPs are synonymous, and do not lead to any changes in the deduced amino acid sequence (Figure 4). The IgG-binding region of SCM obtained from *S. canis* strain G15 does not differ from the *scm* sequence of strain G361, strongly suggested that reduced SCM expression has led to the non-binding phenotype of strain G15. In contrast, strain G17 is characterized as *scm*-negative but, similar to G15, showed an intermediate IgG binding capacity in radioactive binding experiments and FACS analysis. Again, two explanations are reasonable. G17 might harbor a *scm* allele which is not targeted by the all-*scm* PCR or, alternatively, another protein expressed on the surface of G17 harbors IgG binding capacity. Whole genome sequencing and subsequent bioinformatic analysis would be a proper strategy for explaining this phenomenon.

The central position of IgG-binding sites is a common feature of streptococcal IgG-Fc receptors (Nitsche-Schmitz et al., 2007; Meehan et al., 2009; Smeesters et al., 2010), which we also observed for SCM (Figure 3A). As mentioned above, M- and M-like proteins are characterized by a fibrillar structure and a modular organization that roughly divides them into a (hyper-) variable N-terminal part and a relatively conserved C-terminal part. Variability in the N-terminus might be expected due to continuous interaction with the host's immune system that inevitably shapes the M protein's amino acid sequence and constitutes, therefore, one of the numerous immune evasion mechanisms of streptococci. The C-terminus, on the other hand, is usually protected by the bacterial capsule, which prevents direct contact between this part of the M protein and the host's immune system. As a consequence, the amino acid sequences of streptococcal M proteins are highly conserved. The central region of IgG-binding sites in M proteins appear to be contradictory to its role as an immune evasion mechanism, since a direct contact with the host's immunoglobulins is abrogated. However, capsule expression is a very dynamic process and can vary depending on environmental and stress conditions, such as nutrient availability or antibiotic treatments (Hammerschmidt et al., 2005; Willenborg et al., 2011; Haas and Grenier, 2016; Kietzman et al., 2016). Under nutrient-rich conditions, capsule expression is highly upregulated which is consistent with numerous studies characterizing the bacterial capsule as the main virulence factor of streptococci in the blood (Wu et al., 2014). In contrast, at tissue sites, where nutrient-availability is likely restricted, capsule expression is downregulated, leading to demasking of surface-anchored proteins which are otherwise hidden. Mucosal surfaces constitute such tissue sites and *S. canis* is a typical constituent of the respiratory and gastrointestinal microbiota from dogs and cats (Devriese et al., 1992). The microbiome is a complex entity shaped by numerous host-intrinsic and-extrinsic factors. To be able to occupy a specific niche in the host's microbiota, *S. canis* has not only to compete for nutrients with other resident bacteria, but also has to resist the attacks of the immune system which are mainly executed by secreted Igs. In a recent study, Nordenfelt and colleagues nicely demonstrated the importance of the streptococcal IgG-Fc receptors *in vivo* (Nordenfelt et al., 2012). The authors found that at mucosal surfaces (in the throat), IgG is, indeed, bound to the surface of GAS in a non-opsonic manner. In contrast, the orientation of surface-bound antibodies is reversed in the blood, which leads to a Fab-mediated bacterial opsonization and subsequent phagocytic killing (Nordenfelt et al., 2012).

The strict correlation between a *scm*-positive genotype and the ability to interact with IgG strongly suggests that SCM is the only IgG receptor in *S. canis*. This was an unexpected finding, as its closest relatives, GAS and SDSE, usually contain more than one IgG-Fc-binding protein (Bjorck and Kronvall, 1984; Akesson et al., 1994; Frick et al., 1994, 1995; Jensen and Kilian, 2012). For example, SDSE harbors FOG and protein G, that both belong to the large family of streptococcal M and M-like proteins. FOG and protein G differ in their ligand specificity. In addition to its strong affinity for fibrinogen, FOG interacts with the human IgG subclasses IgG1, IgG2, and IgG4 (Nitsche-Schmitz et al., 2007). However, protein G binds to albumin and the entire collection of human IgG subtypes (Bjorck and Kronvall, 1984; Wideback and Kronvall, 1987; Egesten et al., 2011). The localization of the binding sites for FOG and protein G on the IgG molecule appears to be either identical or at least in close proximity, since both proteins specifically inhibit each other in competitive binding experiments. Interestingly, the binding of FOG to the Fc part of IgG allows an additional opsonization with the complement component C1q, which is absent in protein G-IgG interactions. Our studies on SCM-IgG interactions clearly demonstrated that, in terms of ligand specificity, SCM resembles the FOG phenotype and binds to the human IgG subclasses 1, 2, and 4 (Table 2). On the other hand, SCM-mediated binding to IgG specifically prevents C1q opsonisation (Figure 5B), which is similar to the scenario described for protein G (Nitsche-Schmitz et al., 2007). It seems that SCM of *S. canis* is a “functional” intermediate between the two IgG-Fc receptors from other GCGS and GAS. However, since sequence analysis of SCM did not reveal any homologies with either the binding region of protein G nor to the IgG-binding site of FOG or any other known streptococcal M proteins (Supplementary Figure 6), we conclude that streptococcal IgG-Fc receptors are of analogous rather than of homologous nature. Interestingly, despite the analogous nature, binding kinetics between streptococcal M proteins and IgG do not vary tremendously. For example, Lewis and colleagues found, that the M protein of *Streptococcus equi* sub. *equi* (FgBP, for fibrinogen-binding protein) binds to recombinant equine IgG4 and IgG7 with high affinity (*K*_a_ values of 6.79^−6^ Mol and 6.96^−6^ Mol, respectively) (Lewis et al., 2008). Nitsche-Schmitz and colleagues determined the dissociation constant of the interaction between human IgG and the S region, the IgG binding site of protein FOG, to 890 pM. However, when the FOG fragment comprised the S-region together with additional flanking regions, the affinities for IgG increased significantly (Nitsche-Schmitz et al., 2007). We observed similar results, when different truncated fragments of SCM were used to study SCM-IgG interactions (Figure 3B, Supplementary Figure 2). A definitive reason is unknown but it can be assumed that regions adjacent to the IgG binding site might have a certain impact on the protein stability or tertiary structure, respectively. Nevertheless, our data adds another piece of evidence supporting the hypothesis of Frick and colleagues, who postulated a convergent evolution among (streptococcal) IgG binding proteins (Frick et al., 1992).

It is interesting to note that the genome of our reference strain *S. canis* G361 (unpublished data), as well as the genome of a publically available *S. canis* strain (NZ_AIDX00000000), both contain an open reading frame with the highest homology to the N-terminus of protein G of SDSE. The resulting protein is almost identical to protein DG12, which was characterized by Sjöbring in the early 1990s (Sjobring, 1992). Protein DG12 was extracted from a group G streptococcus that was isolated from a cow (strain DG12). Since *S. canis* is a classical causative agent of bovine mastitis, it might be assumed that strain DG12, which Sjöbring has classified as “group G streptococcus of bovine origin” is actually *S. canis*. However, similar to protein G, protein DG12 specifically interacts with human albumin, but has entirely lost its ability to bind to IgG. Subsequent *in silico* analyses of protein DG12 (or the *S. canis*' protein G homolog, respectively) revealed the presence of the GA modules facilitating albumin-binding in protein G, but also a complete lack of the C-terminal C repeats which mediate IgG-Fc binding. Maintaining albumin-binding activity might be advantageous for *S. canis*, since it promotes acquisition and uptake of nutrients, such as fatty acids (Malmström et al., 2012), but also facilitates antibacterial activity by interacting with the chemokine MIG/CXCL9 (Egesten et al., 2011). However, the question as to why *S. canis* has lost one of its Fc receptors crucial for colonization of mucosal surfaces, is beyond the scope of this study.

In summary, we present evidence for an additional feature of the *S. canis* M protein SCM. Its role as an IgG-Fc receptor places it within the large family of streptococcal M- and M-like proteins which function as a bacterial receptors of multiple host proteins. Its characterization as an M protein was previously established due to its plasminogen binding activity. Whether the non-opsonic binding to IgG or the immobilization of plasminogen, or a synergistic action of both (Figure 2) constitutes the primary anti-phagocytic mechanism, should be the subject of future studies.

## Author contributions

SB contributed to experimental assay design and preparation of the manuscript. IE performed sequencing and phylogenetic analysis of the IgG-binding site and contributed to the preparation of the manuscript. TK performed surface-plasmon resonance analysis. OG performed FACS analysis. SH contributed to experimental assay design and to discussion. MR performed EM studies and significantly contributed to manuscript preparation and discussion. MF designed and performed experimental assays and wrote the manuscript.

## Funding

SB received funding by the DFG (Be 4570/4-1). MF received support by the Freie Universität Berlin within the Excellence Initiative of the German Research Foundation. This study was in part supported by Niedersachsen-Research Network on Neuroinfectiology (N-RENNT) of the Ministry of Science and Culture of Lower Saxony, Germany, to MF.

### Conflict of interest statement

The authors declare that the research was conducted in the absence of any commercial or financial relationships that could be construed as a potential conflict of interest.

## Supplementary material

Supplementary Figure 1 **Cloning strategy for truncated SCM fragments by an inverse PCR approach**. The mature SCM protein is depicted as a His-tag fusion construct in plasmid *p*QE30. Arrows indicate oligonucleotides with arrowheads pointing to the direction of transcription. Oligonucleotides comprise an *Eco*RV restriction site at their C-terminus. For SCM fragments with truncations in the N-terminus, a common forward primer was designed which is localized downstream of the stop codon. Accordingly, truncated C-terminal fragments were generated with a reverse primer located in the His-tag region. For construction of the deletion fragment KO173225, internal primers were used.

Supplementary Figure 2 **Localization of the IgG-binding site on SCM**. A collection of different truncated fragments of SCM were immobilized in 96-well plate in concentration-dependent manner. Binding was studied using a polyclonal HRP-conjugated rabbit anti-goat antiserum.

Supplementary Figure 3 **Dot Blot analysis to narrow down the IgG-binding site of SCM**. Different truncated fragments of SCM were spotted in a concentration series onto a PVDF membrane. Binding was evaluated using HRP-conjugated rabbit anti-goat antibodies.

Supplementary Figure 4 **Phylogenetic distances of the sequence alignment of the IgG-binding region of SCM and other M proteins and the S region of FOG (AY600861)**. The alignment of the IgG-binding region of SCM and other M proteins includes the same sequences as used for the alignment in Figure 4. Additionally, the IgG-binding region (S region) of FOG is included into the alignment to underline the vast difference (sequence identities of 48.3–49.9%) between the IgG-binding sequence region of SCM and FOG, despite the known IgG-binding capacity of both proteins.

Supplementary Figure 5 **Evaluation of BIAcore sensograms via Global Fit according to 1:1 Langmuir binding model as offered by BIA evaluation software**. Table revealed chi^2^-values and kinetic parameter.

Supplementary Figure 6 **Phylogenetic distances of the sequence alignment of M protein types harbored by streptococcal reference strains additionally compared to the S region of the FOG protein of group G *Streptococcus* sp. (AY600861 S region)**. Sequences of M proteins were used of the following reference strains: PAB549986—*Streptococcus dysgalactiae* subsp. *equisimilis* strain: RE214 (stg2078.0 type); FJ531841—*Streptococcus* sp. “group G” isolate B11 (stCK401.3 type); DQ010932—*Streptococcus* sp. NS31 (stC5344.1 type); AB549993—*Streptococcus dysgalactiae* subsp. *equisimilis* strain RE377 (stG10.0 type); JX028757—*Streptococcus pyogenes* (stCK401 type); JX028646—*Streptococcus* (emm55 type); KC978839—*Streptococcus pyogenes* strain GLS469 (emm55 type). Differences vary between 52.4 and 100% sequence identity.
