# Supplementary figures and images for: SCM, the M Protein of Streptococcus canis Binds Immunoglobulin G
Source: Front Cell Infect Microbiol. 2017 Mar 28;7:80. doi: 10.3389/fcimb.2017.00080 (PMC5368172; doi:10.3389/fcimb.2017.00080)

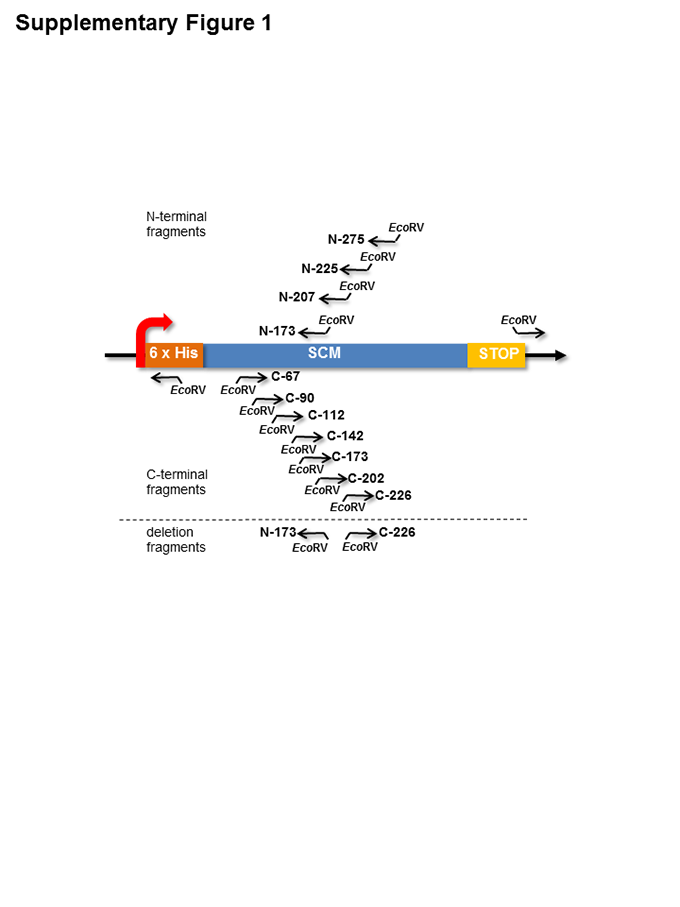

Supplement: Supplementary Figure 1 — Cloning strategy for truncated SCM fragments by an inverse PCR approach. The mature SCM protein is depicted as a His-tag fusion construct in plasmid pQE30. Arrows indicate oligonucleotides with arrowheads pointing to the direction of transcription. Oligonucleotides comprise an EcoRV restriction site at their C-terminus. For SCM fragments with truncations in the N-terminus, a common forward primer was designed which is localized downstream of the stop codon. Accordingly, truncated C-terminal fragments were generated with a reverse primer located in the His-tag region. For construction of the deletion fragment KO173225, internal primers were used. [file Image1.TIF]

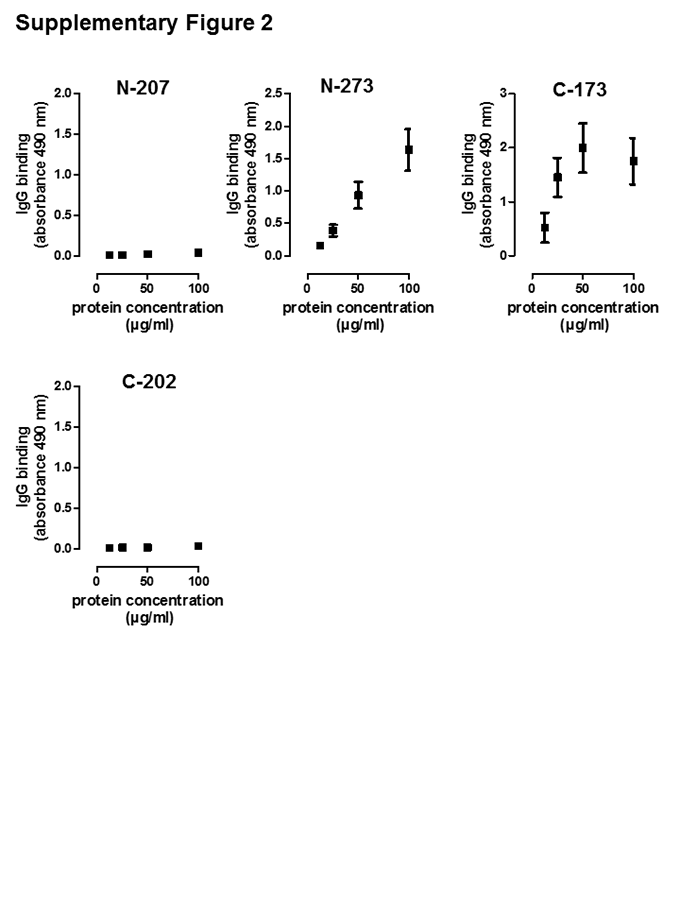

Supplement: Supplementary Figure 2 — Localization of the IgG-binding site on SCM. A collection of different truncated fragments of SCM were immobilized in 96-well plate in concentration-dependent manner. Binding was studied using a polyclonal HRP-conjugated rabbit anti-goat antiserum. [file Image2.TIF]

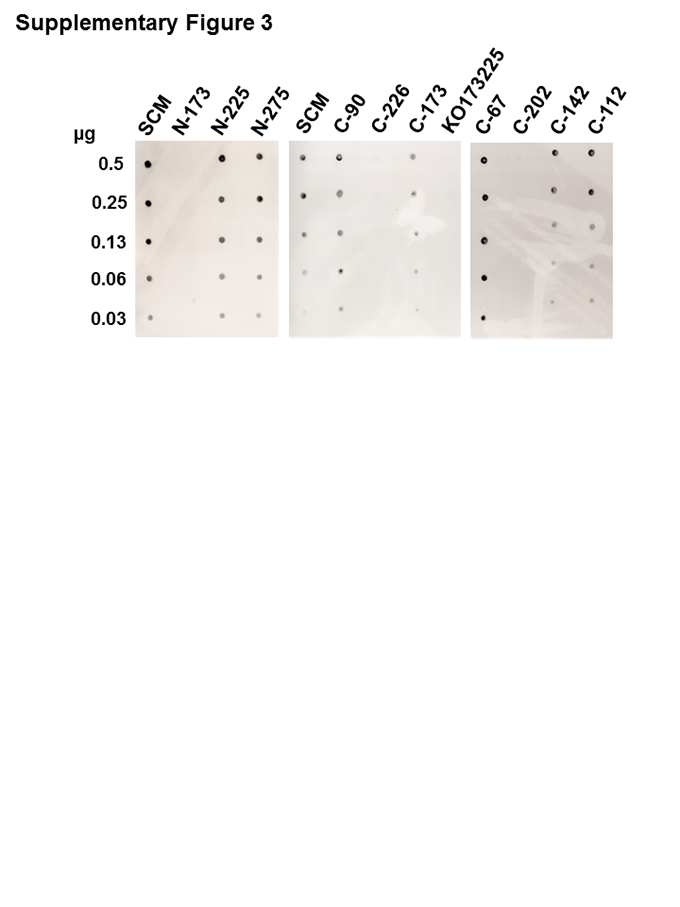

Supplement: Supplementary Figure 3 — Dot Blot analysis to narrow down the IgG-binding site of SCM. Different truncated fragments of SCM were spotted in a concentration series onto a PVDF membrane. Binding was evaluated using HRP-conjugated rabbit anti-goat antibodies. [file Image3.TIF]

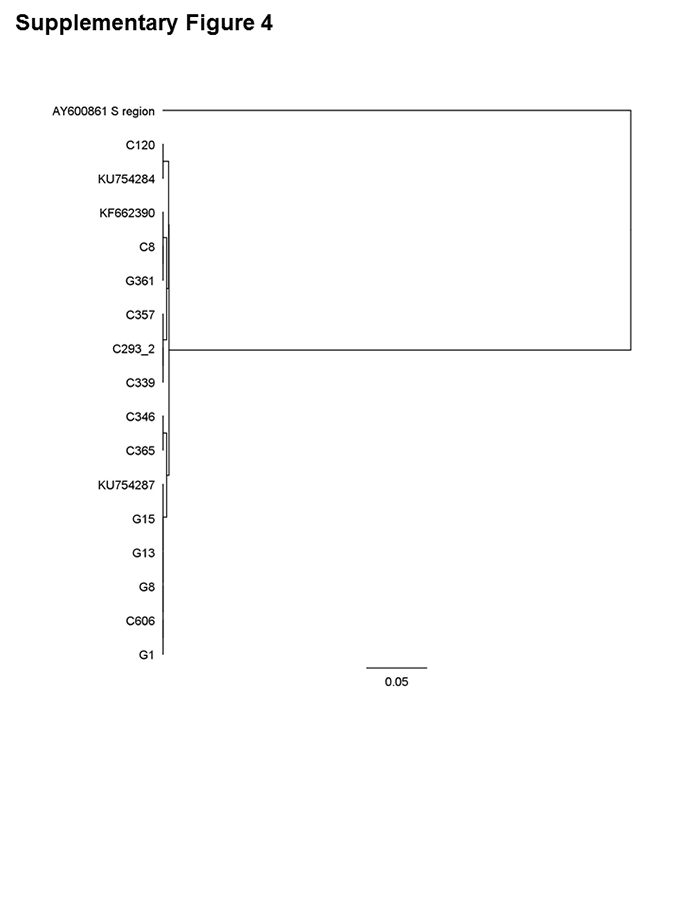

Supplement: Supplementary Figure 4 — Phylogenetic distances of the sequence alignment of the IgG-binding region of SCM and other M proteins and the S region of FOG (AY600861). The alignment of the IgG-binding region of SCM and other M proteins includes the same sequences as used for the alignment in Figure 4. Additionally, the IgG-binding region (S region) of FOG is included into the alignment to underline the vast difference (sequence identities of 48.3–49.9%) between the IgG-binding sequence region of SCM and FOG, despite the known IgG-binding capacity of both proteins. [file Image4.TIF]

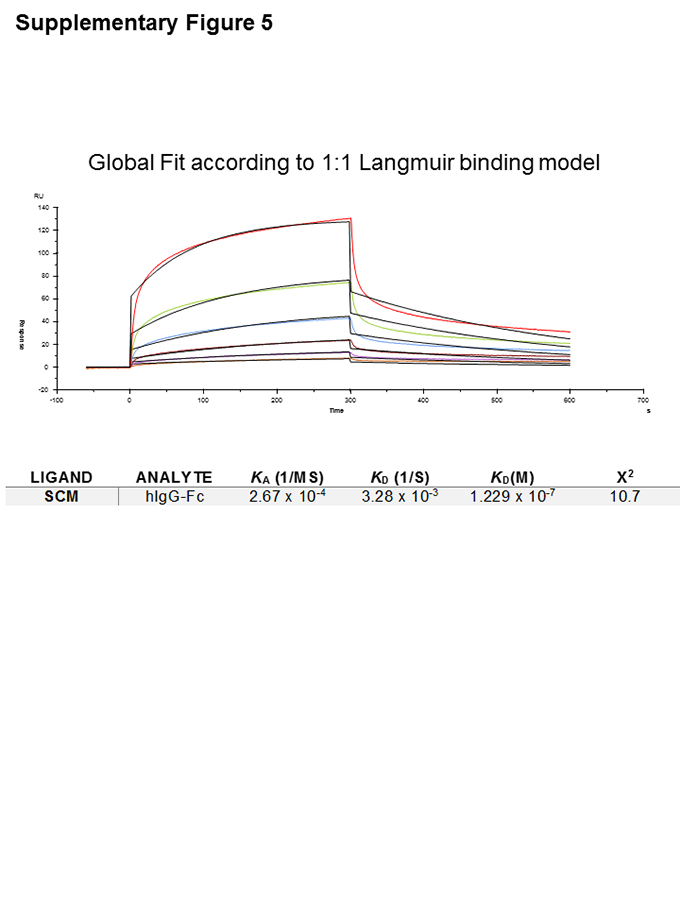

Supplement: Supplementary Figure 5 — Evaluation of BIAcore sensograms via Global Fit according to 1:1 Langmuir binding model as offered by BIA evaluation software. Table revealed chi2-values and kinetic parameter. [file Image5.TIF]

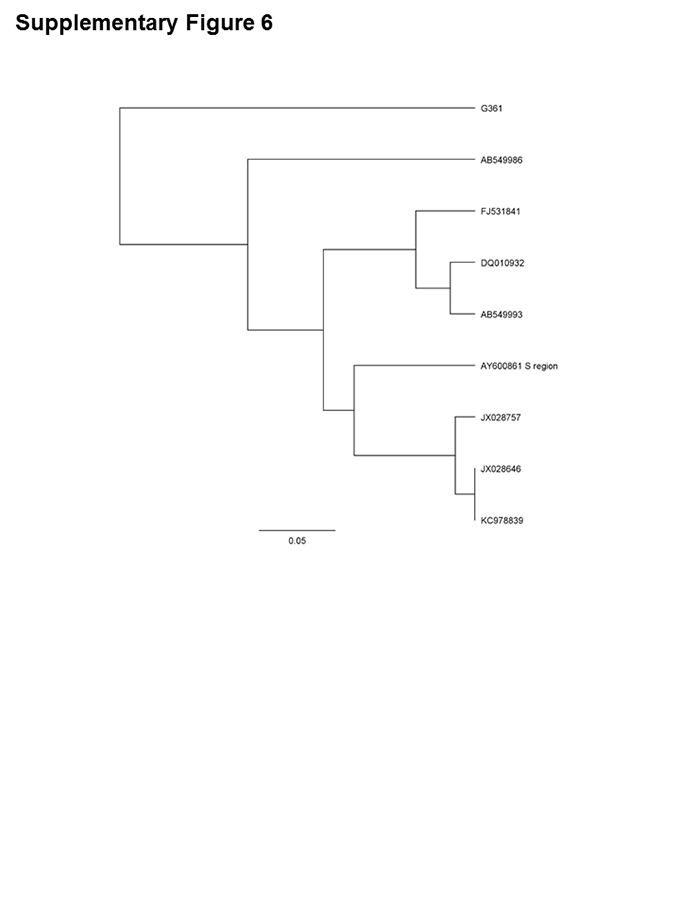

Supplement: Supplementary Figure 6 — Phylogenetic distances of the sequence alignment of M protein types harbored by streptococcal reference strains additionally compared to the S region of the FOG protein of group G Streptococcus sp. (AY600861 S region). Sequences of M proteins were used of the following reference strains: PAB549986—Streptococcus dysgalactiae subsp. equisimilis strain: RE214 (stg2078.0 type); FJ531841—Streptococcus sp. “group G” isolate B11 (stCK401.3 type); DQ010932—Streptococcus sp. NS31 (stC5344.1 type); AB549993—Streptococcus dysgalactiae subsp. equisimilis strain RE377 (stG10.0 type); JX028757—Streptococcus pyogenes (stCK401 type); JX028646—Streptococcus (emm55 type); KC978839—Streptococcus pyogenes strain GLS469 (emm55 type). Differences vary between 52.4 and 100% sequence identity. [file Image6.TIF]
